# Beyond Light: Insights Into the Role of Constitutively Photomorphogenic1 in Plant Hormonal Signaling

**DOI:** 10.3389/fpls.2019.00557

**Published:** 2019-05-16

**Authors:** Wenjing Wang, Qingbin Chen, José Ramón Botella, Siyi Guo

**Affiliations:** ^1^ Key Laboratory of Plant Stress Biology, School of Life Sciences, Henan University, Kaifeng, China; ^2^ Department of Biology and Food Science, Shangqiu Normal University, Shangqiu, China; ^3^ State Key Laboratory of Cotton Biology, School of Life Sciences, Henan University, Kaifeng, China; ^4^ Plant Genetic Engineering Laboratory, School of Agriculture and Food Sciences, The University of Queensland, Brisbane, QLD, Australia

**Keywords:** light signaling, COP1, photomorphogenesis, skotomorphogenesis, plant hormone

## Abstract

Light is an important environmental factor with profound effects in plant growth and development. Constitutively photomorphogenic1 (COP1) is a vital component of the light signaling pathway as a negative regulator of photomorphogenesis. Although the role of COP1 in light signaling has been firmly established for some time, recent studies have proven that COP1 is also a crucial part of multiple plant hormonal regulatory pathways. In this article, we review the available evidence involving COP1 in hormone signaling, its molecular mechanisms, and its contribution to the complicated regulatory network linking light and plant hormone signaling.

## Introduction

Light is an important environmental factor with a profound effect on plant growth and development (Kami et al., 2010). In response to ambient light conditions, plants employ two different developmental programs: skotomorphogenesis and photomorphogenesis. Plants grown in darkness display characteristic-elongated hypocotyls, closed cotyledons, and a pronounced apical hook, while the opposite growth pattern occurs under light conditions (Nemhauser and Chory, 2002; Quail, 2002). Plants have evolved multiple photoreceptors to perceive and distinguish a broad spectrum of light wavelengths (Franklin et al., 2005), including phytochromes (PHYs) that sense red light and far-red light, cryptochromes (CRYs) and phototropins (PHOTs) that perceive blue light, as well as UV resistance locus 8 (UVR8) that responds to UV-B (Christie, 2007; Yu et al., 2010; Chen and Chory, 2011; Tilbrook et al., 2013). Photoreceptor activation by light suppresses the activity of constitutively photomorphogenic1 (COP1) (Podolec and Ulm, 2018), an E3 ubiquitin ligase, which ubiquitinates a number of transcription factors, thus repressing photomorphogenesis. COP1 is active in dark growth conditions accumulating in the nucleus, while light induces its export out of the nucleus leading to the accumulation of transcription factors and promoting photomorphogenesis (Von Arnim et al., 1997; Hardtke et al., 2000; Seo et al., 2003, 2004; Duek et al., 2004; Lau and Deng, 2012; Xu et al., 2016a; Podolec and Ulm, 2018). Although research on COP1 has been focused on its light signaling functions, there are some reports suggesting the involvement of COP1 in other biological processes, such as flowering time regulation, circadian rhythm, and viral defense (Yu et al., 2008; Jeong et al., 2010; Xu et al., 2016b).

Plant hormones play an essential role in plant development, responses to environmental cues, and are central component in stress signaling pathways (Ohri et al., 2015; Sah et al., 2016; Ullah et al., 2018). Hormones enable the plant to perceive internal and external signals and adapt their development using complex signaling networks. Traditional plant hormones include auxins, ethylene (ETH), brassinosteroids (BRs), gibberellins (GAs), cytokinins (CTKs), abscisic acid (ABA), jasmonic acid (JA), and strigolactone (SL). Previous studies have revealed an involvement of hormonal signaling pathway in the light signaling process (Alabadi et al., 2004; Liang et al., 2012; Shen et al., 2012); however, very little is known about the relationship between their signaling pathways. Recent research have reported the involvement of COP1 in multiple hormonal pathways, especially in the regulation of plant de-etiolation, suggesting that COP1 might be the connecting link between light and a plant hormonal signal pathways (Luo et al., 2010; Liang et al., 2012; Zheng et al., 2017).

This review focuses on the recent progress understanding the role of COP1 in plant physiological functions regulated by plant hormones. We discuss the role of COP1 as an integrator in the cross-talk between light and hormonal signal networks.

## Constitutively Photomorphogenic1 Involvement in Auxin Signaling

Auxins participate in the regulation of stomatal development, apical dominance, and hypocotyl elongation (Gray et al., 1998; Steinmann et al., 1999; Saibo et al., 2003); and play a vital role in the adaptation response to environmental changes (Gallavotti, 2013). Light signals modulate auxin synthesis during plant hypocotyl elongation (Xu et al., 2017). Low red light:far-red light (R:FR) ratios induce hypocotyl and petiole elongation, internode extension, increased leaf angle, and acceleration of flowering (Hardtke et al., 2000; Franklin, 2008; Tao et al., 2008), known as shade-avoidance syndrome (SAS). However, in shade conditions, the *cop1* mutant is unable to induce auxin synthesis in hypocotyls indicating a crucial role for COP1 in this process (Pacin et al., 2016).

**Figure 1:**
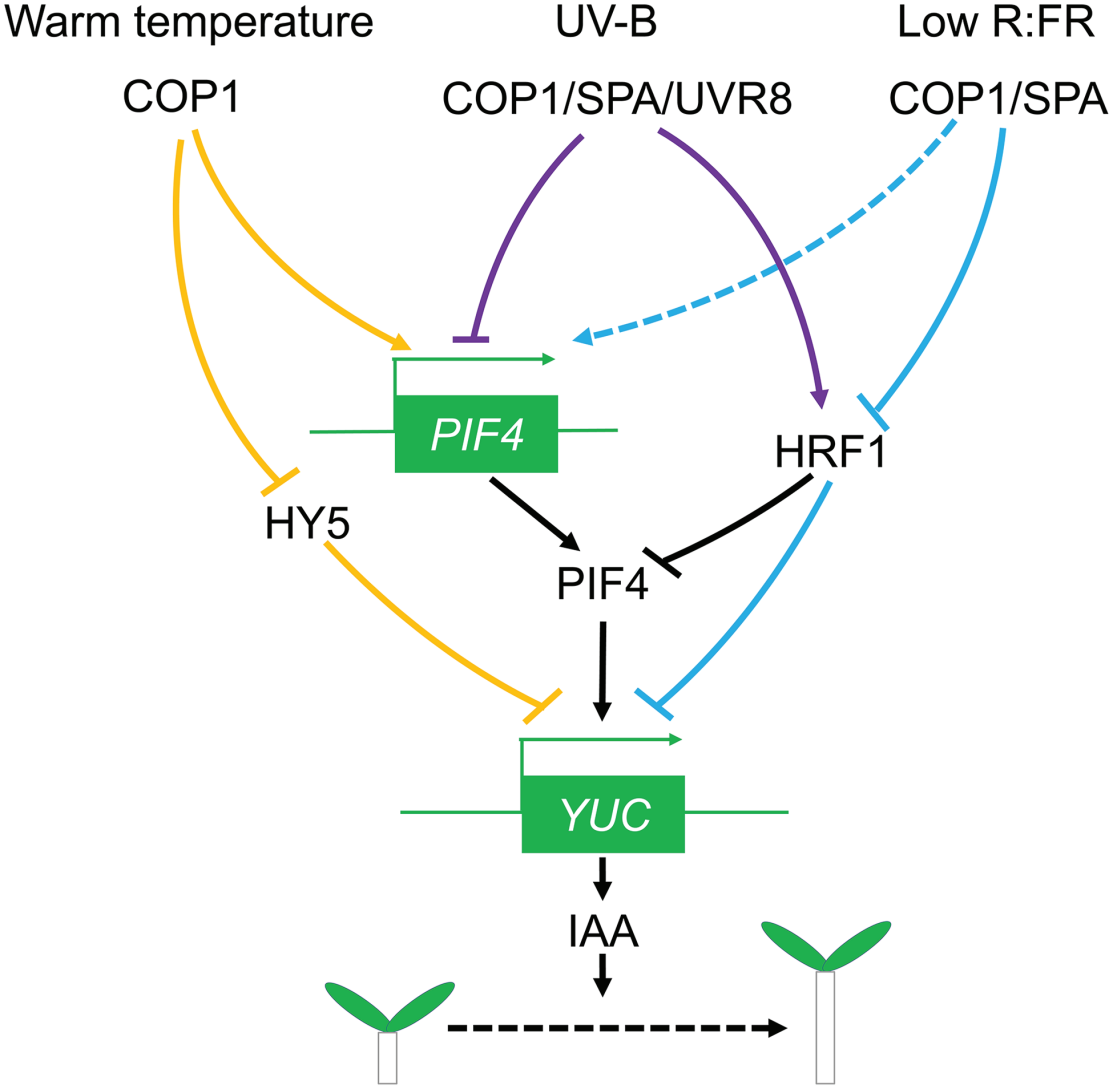
COP1 involvement in auxin-promoted hypocotyl growth. PIF4 can directly promote the expression of YUCCA family genes, thereby increasing auxin content and ultimately enhancing hypocotyl growth. Shade increases COP1/SPA abundance in the nucleus, enhancing *PIF4* transcription and reducing HFR1 levels, which leads to an overall raise in PIF4 transcription factor activity. High temperature also increases COP1 abundance, reducing HY5 levels and enhancing PIF4 activity. UV-B promotes HRF1 accumulation by affecting the activity of the COP1/SPA/UVR8 complex, which in turn inhibits the function of PIF4.

Phytochrome interacting factor (PIF)-deficient mutants can partially restore the features of the *cop1* mutant in the shade, and COP1/SPA can regulate PIF5 stability under red light (Pacin et al., 2016; Pham et al., 2018). PIFs (PIF3, PIF4, PIF5, and PIF7), another class of photomorphogenesis negative regulators, are also involved in the production of auxin in the shade (Lorrain et al., 2008; Pacin et al., 2016). PIFs can directly regulate the expression of auxin synthesis genes. For instance, binding sites for PIF5 are present in the promoters of *YUC5, YUC8*, and *YUC9*, while PIF7 can directly bind to the *YUC8* and *YUC9* promoters (Hornitschek et al., 2012; Li et al., 2012). COP1 may affect PIFs indirectly *via* its control of HFR1, a substrate of COP1, that can block the binding of PIFs to their target genes (Lau and Deng, 2012; Xu et al., 2017). Shade promotes the degradation of HFR1 by COP1 providing a possible mechanism linking COP1, PIF function, and shade avoidance (Pacin et al., 2016). SPA is likely involved in this process since SPA-deficient mutants also exhibit SAS defects similar to *cop1* mutants (Rolauffs et al., 2012). The combined data suggests that COP1 acts mainly as an E3 ubiquitin ligase in SAS. Results published in recent studies have led to the hypothesis that, in *Arabidopsis*, there is probably a COP1/SPA-HRF1-PIFs-YUCCA-shade avoidance pathway, which induces auxin production and thus promotes hypocotyl growth (Figure 1).

Aside from the direct effect on auxin synthesis, light signals can also mediate auxin regulation by temperature (Koini et al., 2009; Sun et al., 2012; Delker et al., 2014). High temperature promotes hypocotyl elongation by stimulating auxin synthesis, and *cop1* mutants are deficient in this response (Park et al., 2017). The high temperature induction of *YUC8* is absent in *cop1* mutants, while overexpression of COP1 results in high levels of *YUC8* expression (Gangappa and Kumar, 2017). Similar to COP1, PIFs also participate in the high-temperature stimulation of auxin synthesis. High temperature induces PIF4 expression and enhances PIF4 binding to the *TAA, CYP79B2,* and *YUC8* promoters, thereby increasing auxin synthesis (Koini et al., 2009; Sun et al., 2012; Di et al., 2016). High temperature-induced upregulation of PIF4 is weakened in *cop1* mutants while overexpression of COP1 results in strong upregulation of PIF4 (Gangappa and Kumar, 2017). Thus, COP1 may be involved in high temperature-induced auxin synthesis through its regulation of PIF4 expression in *Arabidopsis*. HY5 is a major transcription factor in photomorphogenesis and a critical ubiquitination substrate for COP1 in the nucleus. HY5 can compete with PIFs binding to the E/G-box elements of the auxin-producing *YUC8* promoter (Chen et al., 2013; Gangappa and Kumar, 2017), but high temperatures can reduce its binding ability. Since high temperature induces COP1 accumulation in the nucleus (Park et al., 2017), it is possible that the temperature-dependent nuclear accumulation of COP1 results in reduced levels of HY5, relieving the competition with PIF4 in the *YUC8* promoter and facilitating auxin synthesis and hypocotyl growth.

On the other hand, plants exposed to sunlight receive high levels of UV radiation and are likely to experience higher temperature. UV-B promotes the binding of the photoreceptor UVR8 to COP1 decreasing the ubiquitination activity of COP1, and reducing *PIF4* expression levels. In addition, UV-B increases HFR1 stability and the competition with PIF4 for the binding to the *YUC8* promoter, thereby reducing auxin synthesis and inhibiting hypocotyl elongation (Hayes et al., 2017). This may be an indication that COP1 uses multiple mechanisms to affect high temperature-induced auxin synthesis.

COP1 participates not only in the regulation of auxin synthesis but also in polar auxin transport in plants (Zhao et al., 2001; Esmon et al., 2006; Tao et al., 2008; Sassi et al., 2012). Root growth is dependent on the existence of an auxin concentration gradient, controlled by the PIN-FORMED (PIN) efflux carriers *via* control of polar auxin transport. Loss of COP1 function leads to attenuation of light-induced root elongation (Wisniewska et al., 2006), suggesting a link between COP1 and the auxin concentration gradient. PIN1 is involved in light-induced root elongation (Vernoux et al., 2000) and its expression is upregulated in *cop1* mutants (Sassi et al., 2012). PIN2 also participates in root growth modulation under light and although its expression levels are not altered in *cop1* mutants, its stability is increased (Luschnig et al., 1998; Blilou et al., 2005). Similar to *cop1pin1*, *cop1pin2* double mutants exhibit shorter roots than that of *cop1* (Sassi et al., 2012). Hence, the effect of COP1 on polar auxin transport is probably dependent on multiple factors including control of gene expression and changes in PIN stability. Whether PIN2 is a substrate for COP1 is not known.

## Constitutively Photomorphogenic1 Involvement in Ethylene Signaling

The gaseous plant hormone, ethylene, acts as a pivotal mediator in the coordination of growth, defense, and survival in response to environmental challenges (Yoo et al., 2009; Ullah et al., 2018). Most ethylene-associated growth and development processes depend on light signaling. For instance, COP1 plays a crucial role during the process by which ethylene reverses the inhibition of seed germination under salt stress. Salt stress causes an increase of cytoplasmic GUS-COP1, which is reduced by ACC (the precursor of ethylene) treatment (Yu et al., 2016), suggesting that the increase in nuclear localization of COP1 promoted by ethylene is important for the reversal of germination suppression under salt stress. HY5 can upregulate *ABI5* expression, resulting in repression of seed germination (Chen et al., 2008). Therefore, ethylene-induced nuclear import of COP1 may decrease HY5 content, reducing *ABI5* expression, and thus relieving the suppression effect of salt stress on seed germination.

**Figure 2:**
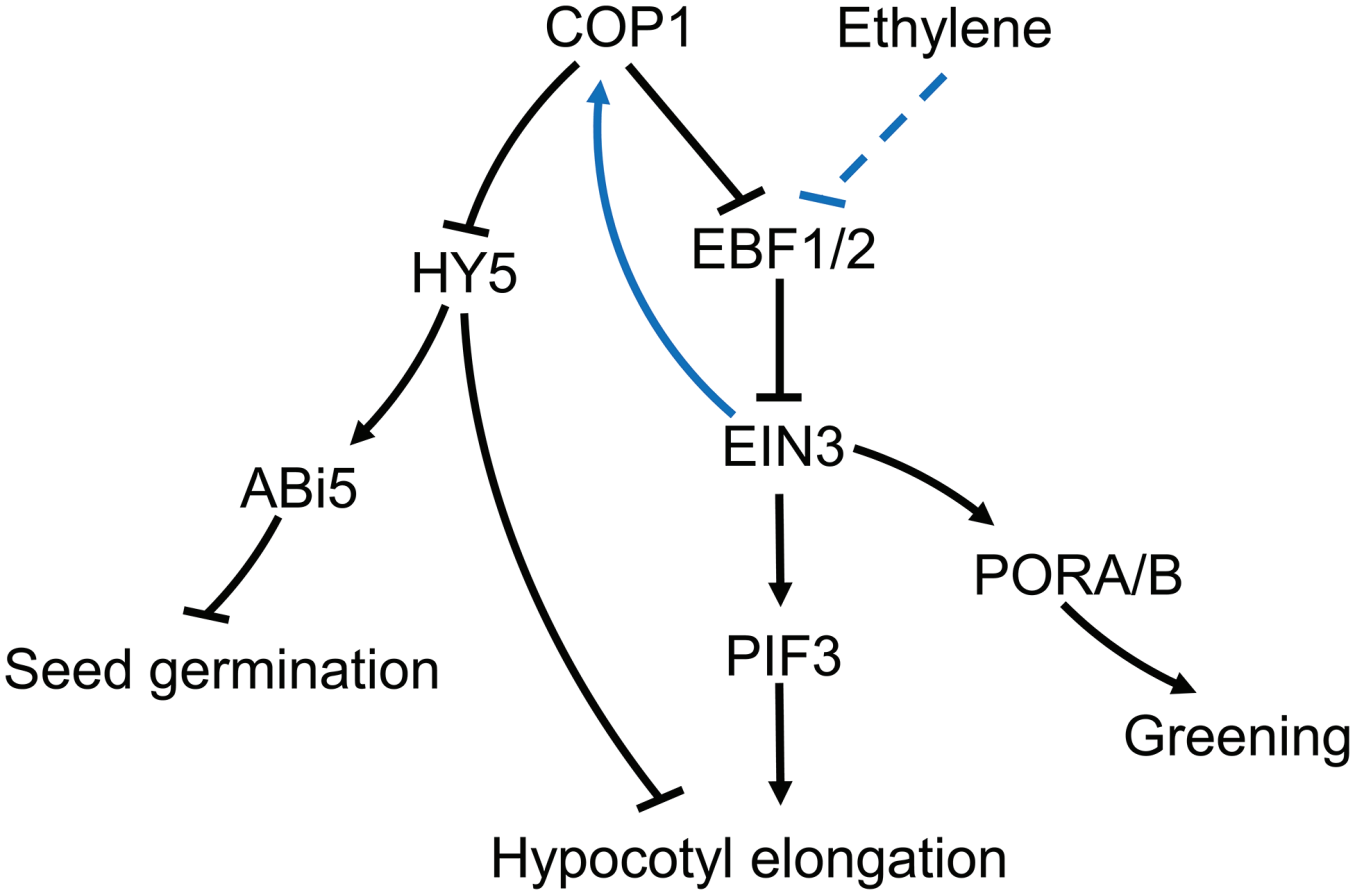
Putative roles for COP1 in the plant’s ethylene signaling pathway. During exposure to light, ethylene prevents degradation of EIN3 *via* EBF1/2. Increased EIN3 levels promote the nuclear import of COP1. Nuclear localized COP1 reduces levels of the transcription factor HY5, thus supporting hypocotyl growth under the light in *Arabidopsis*. At the same time, the increased levels of COP1 in the nucleus can further weaken EBF1/2 and increase EIN3 levels (Shi et al., 2016b). COP1-mediated reduction in HY5 levels prevents ABI5 accumulation, which relieves the salt stress inhibition of seed germination. COP1 effect on EIN3 can affect PORA/B expression promoting cotyledon greening upon light exposure. COP1 can also directly affect cotyledon greening in an ethylene independent manner.

Ethylene promotes hypocotyl growth in *Arabidopsis* under light, but suppresses growth in the dark (Yu and Huang, 2017). However, in *cop1-4* mutants, ethylene can promote hypocotyl growth in the dark, a process that can be suppressed by NPA, an IAA polar transport inhibitor (Liang et al., 2012). Under light, ethylene increases COP1 concentration in the nucleus, reduces HY5 stability, and promotes hypocotyl growth (Yu et al., 2013). Nuclear accumulation of COP1 is dependent on ethylene insensitive 3 (EIN3) (Yu et al., 2013). The transcription factor, EIN3, is a positive regulator of the ethylene response and a substrate of the EIN3-binding F box protein 1/2 (EBF1/2). EBF1/2 induces the degradation of EIN3 by the ubiquitination pathway in the absence of ethylene (Gagne et al., 2004; Chen et al., 2005). It has been reported that COP1 may promote EIN3 stability through ubiquitination of EBF1/2 (Shi et al., 2016a,b), although the mechanism controlling the ubiquitination of EBF1/2 by COP1, and the way in which ethylene influences this process, is not fully understood. The COP1-induced increase in EIN3 stability can increase *PIF3* transcription and promote hypocotyl growth under light (Zhong et al., 2014). In addition, ethylene can accelerate EIN3 accumulation in *cop1-4* mutants, indicating that ethylene and COP1 independently affect EIN3 stability through EBF1/2 (Yu and Huang, 2017). Overall, the accumulated data suggests the existence of an ethylene-EBF1/2-EIN3-COP1-HY5 pathway in plants, which promotes hypocotyl growth of *Arabidopsis thaliana* under light conditions (Figure 2).

Compared with the available knowledge on the ethylene involvement in light-induced hypocotyl growth, there is scant information on the suppression effects in the dark. In the dark, COP1 reduces the stability of HY5, the photomorphogenesis-positive regulatory factor, and promoting hypocotyl growth (Osterlund et al., 2000). However, ethylene can suppress this process by enhancing transcription of the *ethylene response factor 1 (ERF1)* and prevent hypocotyl growth by regulating EIN3. Meanwhile, EIN3 promotes transcription of *Wave-Dampened 5* (*WDL5*), an important component of the ethylene-mediated suppression of hypocotyl growth in the dark (Sun et al., 2015). It has been proposed that the COP1-HY5 and ethylene pathways act antagonistically in the dark. However, the fact that ethylene can promote *cop1-4*’s hypocotyl growth in the dark suggests that COP1 can promote the suppression of hypocotyl growth by ethylene in the dark (Liang et al., 2012). The involvement of COP1 in the suppression of hypocotyl growth by ethylene in darkness needs further research before a clear picture can emerge.

Ethylene plays a crucial role in cotyledon greening upon exposure to light (Zhong et al., 2009). The regulatory component EIN3 can bind to the promoters of the protochlorophyllide oxidoreductases, *PORA* and *PORB*, two key enzymes in the synthesis of chlorophyll (Reinbothe et al., 1996). The cotyledons of *cop1*-*4* mutants have a yellowish appearance, and ethylene can restore greening upon exposure to light. Moreover, over-expression of *EIN3* on a *cop1* mutant background can also restore cotyledon greening. Therefore, COP1 may induce expression of *PORA* and *PORB* through repression of EBF1/2 and accumulation of EIN3 promoting cotyledon greening under light exposure (Figure 2).

## Constitutively Photomorphogenic1 Involvement in Brassinosteroid Signaling

Light can suppress hypocotyl elongation and promote chlorophyll accumulation; however, such processes can be reversed by brassinosteroids (BRs) (Terzaghi and Cashmore, 1995; Luo et al., 2010). Moreover, BR-deficient and insensitive mutants display typical de-etiolation in the dark and show upregulation of numerous light-induced genes (Chory et al., 1991; Li et al., 1996; Song et al., 2009). The molecular mechanisms used by BR to suppress photomorphogenesis and promote skotomorphogenesis have only recently begun to be unraveled with COP1 playing a crucial role (Luo et al., 2010; Shi et al., 2011; Kim et al., 2014).

Light promotes photomorphogenesis by regulating a series of genes containing light-response elements (LREs) in their promoters, including G-box, GATA, and GTI motifs (Terzaghi and Cashmore, 1995; Lau and Deng, 2012). The transcription factor GATA2 binds the GATA motif and is a positive regulator of photomorphogenesis (Luo et al., 2010). Darkness promotes degradation of GATA2 on a COP1-dependent manner, and COP1 can ubiquitinate GATA2 *in vitro* (Luo et al., 2010). In addition to light signaling, GATA2 is also regulated by the transcription factor, brassinazole-resistant 1 (BZR1), involved in the BR signaling pathway in plants (He et al., 2005). In the dark, the activated BZR1 strongly binds to the *GATA2* promoter, preventing transcription of *GATA2*, and thus suppressing photomorphogenesis (Luo et al., 2010). Therefore, GATA2 seems to play a crucial role in the cross-talk between light and BR signaling. COP1-mediated degradation of GATA2 promotes skotomorphogenesis, while BR can suppress its expression and thus accelerate skotomorphogenesis. However, it has not yet been proven whether there is an association between these two different mechanisms.

COP1 is also probably involved in the attenuation of BR signaling by light. Perception of BR at the plasma membrane is performed by the leucine-rich receptor, kinase brassinosteroid insensitive 1 (BRI1) and its chaperone bri1-associated receptor kinase 1 (BAK1) (Li et al., 2002; Nam and Li, 2002; Santiago et al., 2013). Membrane steroid binding protein 1 (MSBP1) suppresses the perception of BR by binding with BAK1 and enhancing the endocytosis of BAK1 (Song et al., 2009). *MSBP1* expression is inhibited in the dark and this suppression reduced in *cop1* mutants (Shi et al., 2011). *MSBP1* expression can be induced by the COP1 substrates, HY5 and HYH under light, and its expression pattern is similar to that of *HY5* (Yang et al., 2005; Sibout et al., 2006; Jiao et al., 2007). Although it remains unclear whether COP1 directly affects the stability of the MSBP1, darkness can lead to a reduction of MSBP1 levels, which can thereby stimulate the transduction of the BR signal, thus promoting skotomorphogenesis. There is no direct evidence suggesting a role for COP1 in the suppression BR signaling by light, but a COP1-HY5-MSBP1 pathway, regulating MSBP1 concentration *via* HY5 is possible.

Moreover, COP1 can directly regulate other critical factors in the BR signaling pathway, such as brassinosteroid insensitive 2 (BIN2), a critical negative regulator of the BR signaling (He et al., 2002; Yin et al., 2002). In the absence of BR, BIN2 phosphorylates brassinazole-resistant 1 (BZR1) and BRI1-EMS-suppressor 1 (BES1) (He et al., 2002; Yin et al., 2002, 2005). It has been recently reported that BIN2 can phosphorylate the photomorphogenesis suppressors PIF3 and PIF4; reducing the stability of PIF3 through degradation by the 26 s proteasome pathway (Bernardo-Garcia et al., 2014; Ling et al., 2017). It is not known whether BIN2 can also phosphorylate other PIFs, but there is evidence that PIFs may be involved in promoting BR signaling in darkness. As a negative regulator of BR responses, BIN2 can reduce skotomorphogenesis by impairing PIF activity, and suppress the function of BR in the dark. In addition to binding with BIN2-PIF3, the COP1/SPA complex can interfere with the phosphorylation of PIF3 by BIN2 (Ling et al., 2017). In this process, BIN2 is not ubiquitinated; however, COP1 may inhibit the function of BIN2 and prevent phosphorylation to maintain skotomorphogenesis.

**Figure 3:**
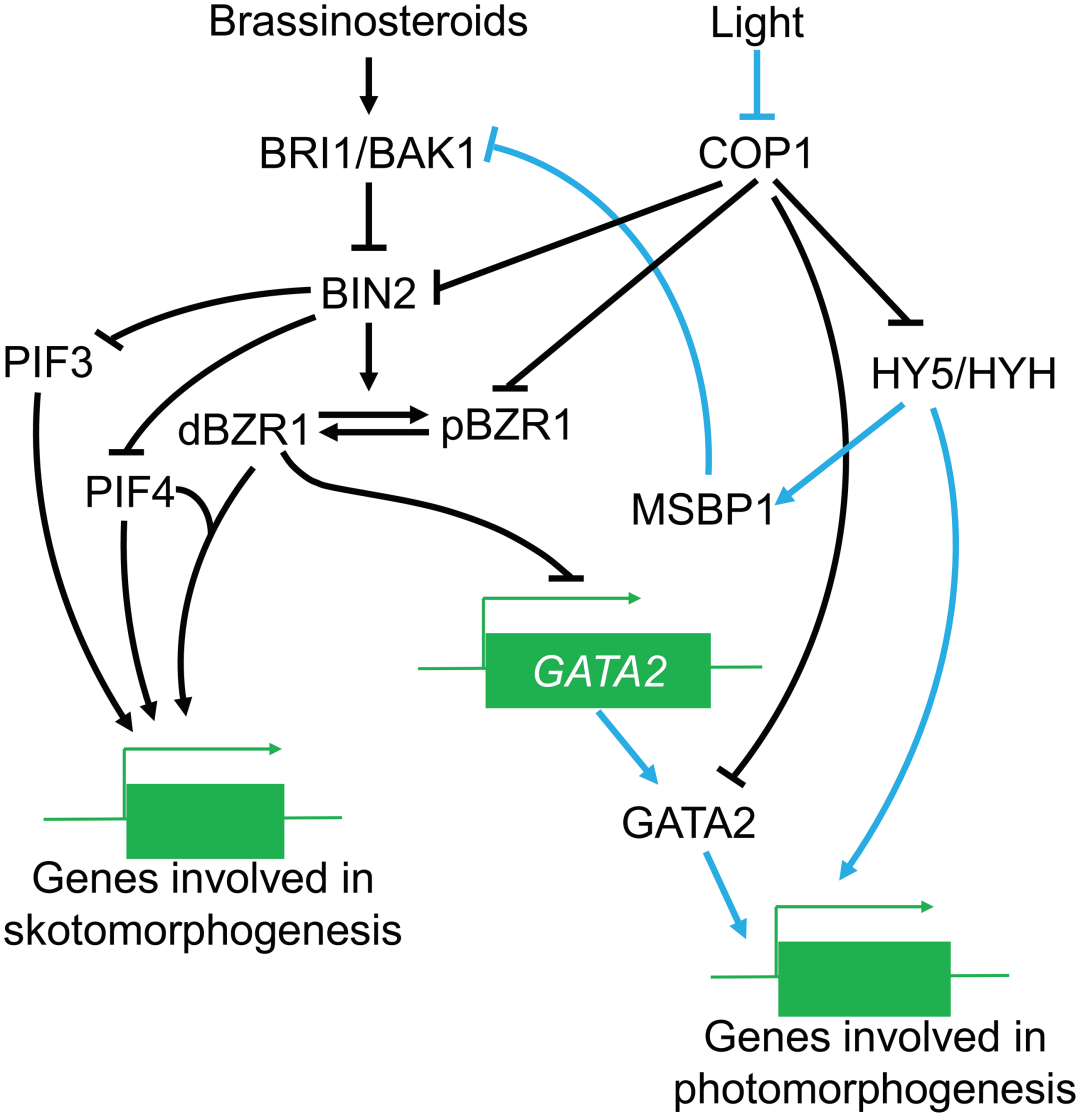
The role of COP1 in the BR signaling pathway. Under the light conditions, COP1 is transported out of the nucleus, leading to enhanced HY5/HYH accumulation and increased expression of genes involved in photomorphogenesis, COP1 also induces MSBP1 accumulation, which can thereby interfere with the function of BRI1/BAK1 and affect the transduction of BR signals. Meanwhile, COP1 suppression of BIN2 and pBZR1 is reversed, positively affecting BR signal transduction while GATA2 is activated to promote photomorphogenesis. In the dark, accumulation of COP1 in the nucleus inhibits the activities of positive photomorphogenesis regulators (such as HY5); on the other hand, COP1 might also enhance the suppression of negative regulators in the BR signal, thus promoting the expression of genes in skotomorphogenesis.

Importantly, COP1 affects both the effects of BIN2 on the BR signal and also regulates the BIN2 phosphorylated substrate, BZR1. BZR1 has two phosphorylation forms with different functions in the BR pathway: phosphorylated (pBZR1) and dephosphorylated BZR1 (dBZR1) (Li et al., 2017a). Phosphorylated pBZR1 can be captured by 14-3-3 proteins and confined to the cytoplasm (Gampala et al., 2007; Ryu et al., 2007); reducing its capacity to bind DNA and reducing BR signaling (Vert and Chory, 2006). Therefore, the pBZR1 acts as an inactive state in the BR response. COP1 can ubiquitinate BZR1 and has a preference for the phosphorylated pBZR1 (Kim et al., 2014) although the molecular basis for this preference is not well understood. There is evidence that the antagonism between light and BR in photomorphogenesis may be potentially dependent on the regulation of the BR signal by COP1. A speculative model of the regulation of BR signaling by COP1 is illustrated in Figure 3.

## Constitutively Photomorphogenic1 Involvement in Gibberellin Signaling

Similar to BR, gibberellins can also suppress photomorphogenesis and promote skotomorphogenesis (Alabadi et al., 2004; Feng et al., 2008). However, the promotion of skotomorphogenesis by GA depends mainly on the endogenous GA content.

In the dark, reduction in GA levels induces de-etiolation in plants; however, this process is impaired in the *hy5* mutant (Alabadi et al., 2008), suggesting an important role for HY5 in the GA control of skotomorphogenesis. PAC, an inhibitor of GA synthesis, decreases GA levels and induces HY5 protein accumulation but does not affect *HY5* transcription levels, similar to the effect of light signaling. It has been shown that neither exogenous GA nor PAC can change the HY5 levels in *cop1-4* mutants (Alabadi et al., 2008). Even though it remains unclear whether PAC treatment in the dark triggers the nuclear export of COP1, the available indirect evidence suggests that the COP1-HY5 pathway is associated with the promotion of skotomorphogenesis by GA. Mutations in *PIF* genes, another photomorphogenesis negative regulator, enhance de-etiolation under PAC treatment (Bauer et al., 2004; Shen et al., 2005; Alabadi et al., 2008) with GA directly enhancing PIFs protein activity (Mazzella et al., 2014), but a direct connection needs to be proved. GA binding to the GA receptor gibberellin insensitive dwarf1 (GID1), induces the degradation of DELLA, a negative regulator of the GA signal (Peng et al., 1997; Willige et al., 2007; Mazzella et al., 2014). DELLA can bind onto PIFs and reduce their activity (Alabadi et al., 2008; de Lucas et al., 2008; Feng et al., 2008); it can also enhances HY5 stability (Alabadi et al., 2008), suggesting that DELLA suppresses GA signal transduction, and is also involved in photomorphogenesis.

**Figure 4:**
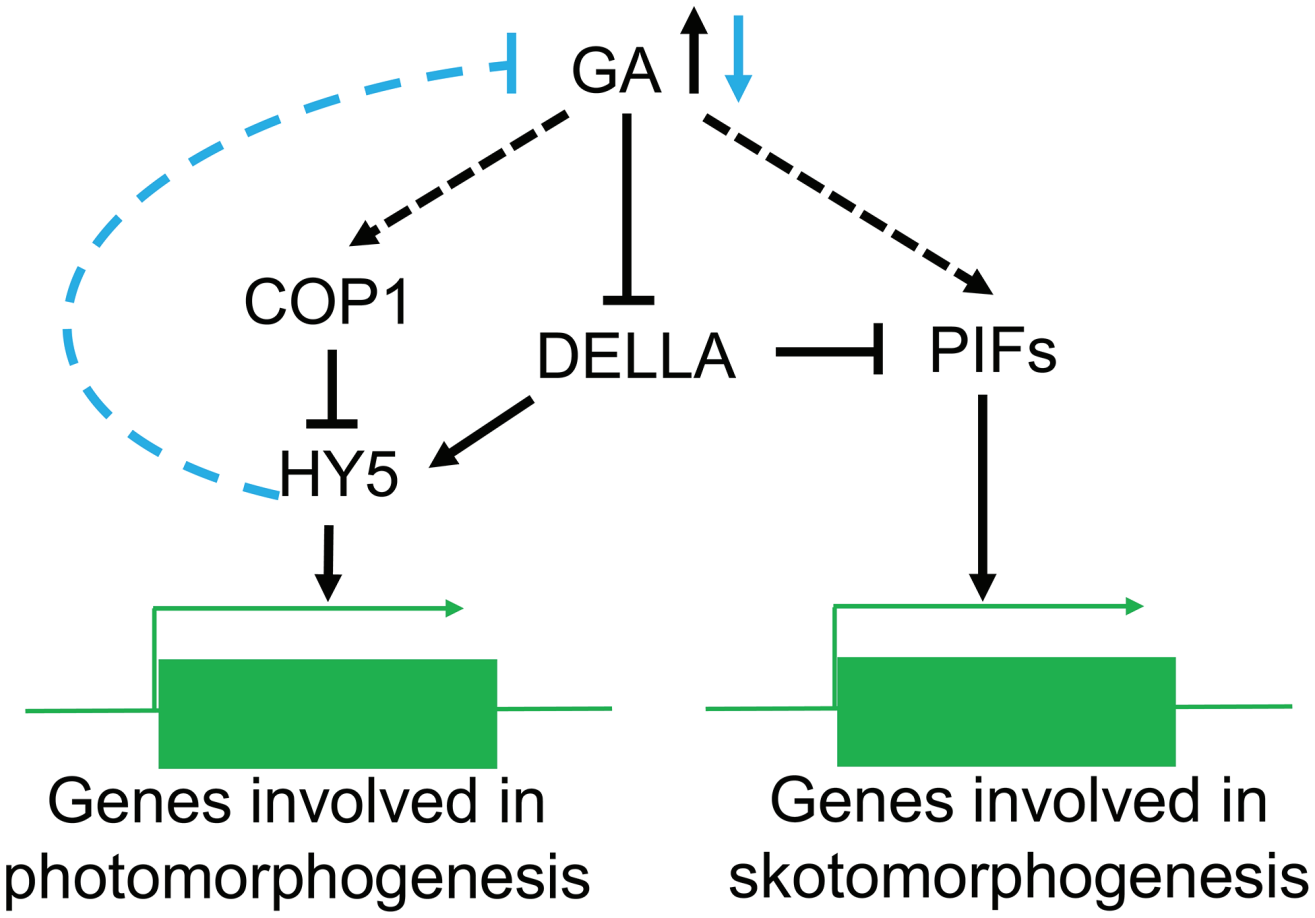
The role of COP1 in the GA signaling pathway. In the dark, high GA levels lead to a reduction of DELLA relieving the suppression of PIFs activity and promoting the expression of genes involved in skotomorphogenesis. At the same time, COP1 can reduce the accumulation of HY5 protein in the nucleus and prevent the expression of photomorphogenesis genes. In the light, confinement of COP1 to the cytoplasm induces accumulation of HY5 and a reduction in the GA levels. The decrease in GA content will also reduce the suppression of DELLA resulting in increased PIFs activity.

GA can suppress photomorphogenesis in the dark, and light reverses this process by decreasing GA content (Weller et al., 2009) in *Arabidopsis thaliana*, pea, and rice (Mazzella et al., 2014). For instance, blue light can reduce GA synthesis by activating CRY1 (Zhao et al., 2007; Vandenbussche et al., 2007a). Mutations in *LONG1*, the homologous gene of HY5 in pea, produce a dramatic impairment in the light regulation of active GA levels and the expression of several GA biosynthetic genes but it does not influence GA signal transduction (Alabadi et al., 2008; Weller et al., 2009). Conversely, the *lip1* mutant (the homologous gene of *cop1* in pea) has lower GA levels in the dark but normal wild-type levels in the light (Sullivan and Gray, 2000; Weller et al., 2009). It has been suggested that HY5 participates in the light-regulated reduction in the GA content, while COP1 does not directly regulate GA biosynthesis during in the light (Figure 4). Nevertheless, the molecular mechanisms controlling HY5 regulation of GA levels are not fully understood.

## Constitutively Photomorphogenic1 Involvement in Jasmonic Acid Signaling

Jasmonic acid (JA) is a vital defense hormone in plants, which plays a crucial role in response to fungal invasion and insect attack (McConn et al., 1997; Nibbe et al., 2002). JA is also involved in responses to environmental stimuli and the regulation of skotomorphogenesis (Huang et al., 2017) inhibiting hypocotyl growth in the dark and promoting de-etiolation (Hsieh and Okamoto, 2014). It has been suggested that JA promotes dark morphogenesis in conjunction with other factors. For instance, JA suppresses the growth of wild-type hypocotyls and the development of cotyledons in the dark, but it has an insignificant effect on *cop1-4* mutant (Zheng et al., 2017).

JA signaling is mainly dependent on the release of MYC transcription factors by jasmonate-zim-domain proteins (JAZs) (Chini et al., 2007). In the absence of JA, JAZ binds and inhibits the MYC transcription factors (Chini et al., 2007; Thines et al., 2007; Hsieh and Okamoto, 2014). JA activates coronatine insensitive 1 (COI1), an E3 ligase, leading to JAZ degradation and the release of MYC2 (Thines et al., 2007). MYC2 can increase HY5 levels during photomorphogenesis (Prasad et al., 2012). Although there is no evidence that HY5 accumulation occurs due to impairment of COP1 activity by MYC2, a recent study has shown that *myc2* mutants cannot restore the *cop1-6* phenotypes in JA responses (Zheng et al., 2017), suggesting that MYC2 can affect the function of COP1. A different study has shown that SPA, an essential binding protein for COP1, is regulated by MYC2 in the JA responses (Gangappa et al., 2010). Therefore, MYC2 might affect the role of the COP1-SPA complex in JA signaling, although the existence of such a regulatory mechanism has not yet been proven. Nevertheless, it has been demonstrated that JA can attenuate the formation of the COP1-SPA complex and affect COP1 function in the nucleus (Zheng et al., 2017). Thus, the existence of a pathway in plants, whereby JA regulates MYC2, and thus changes the function of the COP1-SPA complex promoting photomorphogenesis is speculative but possible.

The *Arabidopsis* far-red (light)-insensitive 219/jasmonate resistant 1 (FIN219/JAR1) gene encodes a jasmonate-amido synthetase, which catalyzes the synthesis of Jasmonyl-isoleucine (JA-Ile), a bioactive form of JA (Staswick and Tiryaki, 2004; Hsieh and Okamoto, 2014). FIN219 also plays a significant role in regulating hypocotyl elongation in the shade (Swain et al., 2017). The *fin219* mutant exhibited longer hypocotyls in the shade than wild type, but it could not restore the shorter hypocotyls of *cop1-6* under shading (Swain et al., 2017), suggesting that FIN219’s inhibition of hypocotyl elongation is dependent on COP1. Although the molecular mechanisms by which FIN219 influences COP1 are not fully known, it has been reported that when plants are in the shade, FIN219 can enhance COP1 accumulation in the cytoplasm. Paradoxically, darkness increases COP1 accumulation in nucleus, while FIN219 can enhance the cytoplasmic accumulation of COP1. This may imply that plants under shade can induce excessive COP1 accumulation in the nucleus through FIN219, and thereby regulate hypocotyl elongation to cope with shading by other plants. Blue light can actively induce binding of FIN219 to COP1 and promote the COP1 export (Chen et al., 2018). However, MeJA can significantly boost the binding of FIN219 and GUS-CCT1 (ectopic expression of the C-terminal domain of CRY1) under blue light, and relieve the inhibition of COP1-SPA activity by CRY1, thereby reducing the nuclear accumulation of HY5 and promoting the growth of hypocotyls. Hence, JA can regulate hypocotyl growth by attenuating FIN219 binding to COP1 under the light, and enhancing COP1 activity (Chen et al., 2018).

**Figure 5:**
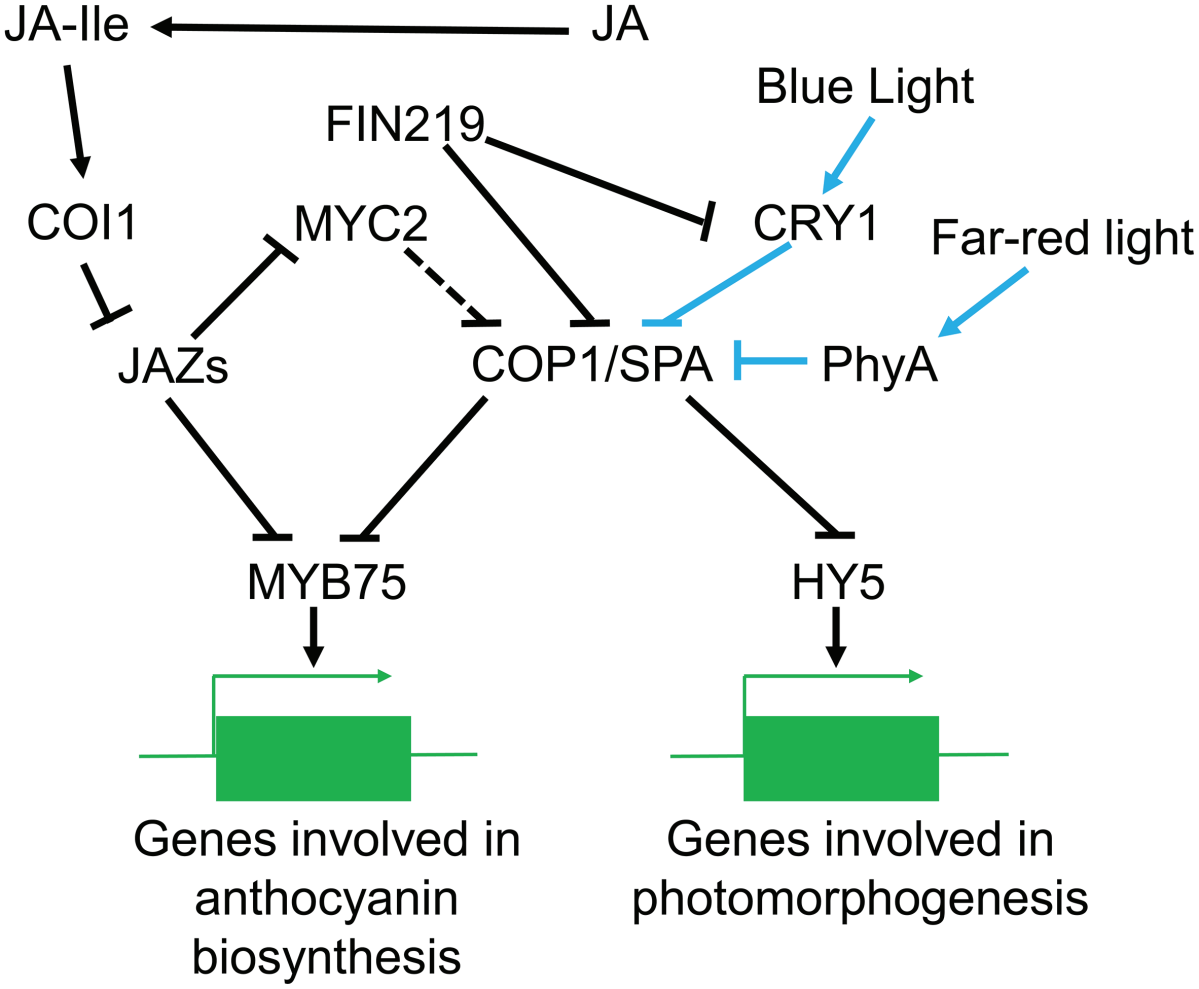
The role of COP1 in JA signaling pathways in plants. When the JA content is low, JAZs inhibit MYC2 function release the of COP1/SPA complex to facilitate skotomorphogenesis. However, with an increased level of JA in the dark, COP1 degrades JAZs proteins and eliminates their inhibition of MYC2, ultimately promoting photomorphogenesis. In the dark, COP1/SPA can suppress synthesis of anthocyanins by promoting degradation of MYB75 in the nucleus. However, far-red light can inhibit the function of the COP1/SPA complex and promote stabilization of MYB75. Meanwhile, JA can promote MYB75 activity, which causes anthocyanin accumulation in the light. In blue light, CRY1 inhibits COP1/SPA activity, while FIN219 increases cytoplasmic COP1 and further weakens COP1/SPA function, thereby promoting photomorphogenesis. MeJA can enhance the role of COP1 by inducing the binding of FIN219 to CRY1, thereby regulating the growth of hypocotyls.

JA can promote anthocyanin accumulation in WT under light, but not in the dark (Qi et al., 2011). A recent study, demonstrated that *cop1* mutants undergo substantial accumulation of anthocyanins in the dark when 5 μM MeJA is applied (Li et al., 2014). The myeloblastosis protein 75 (MYB75), a member of the MYB transcription factor family, can upregulate the critical enzymes in the anthocyanin production pathway, including dihydroflavonol 4-reductase (DFR), leucoanthocyanidin dioxygenase (LDOX), and UDP-GLC: flavonoid 3-o-glucosyltransferase (UF3GT), thereby increasing anthocyanin content (Dooner et al., 1991; Li et al., 2014). MYB75 activity can be suppressed by JAZs, the main repressors in JA signaling. In the light, JA can relieve the inhibition of MYB75 by JAZs, thereby promoting MYB75 activity, while in the dark, COP1 can bind to MYB75 and promote its degradation. Although these two different pathways partially explain how JA regulates anthocyanin content in light and dark conditions, they do not explain why JA cannot weaken COP1’s effect on MYB75 in the dark. Many aspects of the cross-talk between COP1 and JA signaling in the dark have not yet been confirmed. For example, it has been reported that JA can prevent the production of protochlorophyllide (Pchlide) in the dark (Sineshchekov et al., 2004), while COP1 is essential for the accumulation of protochlorophyllide oxidoreductase A (PORA) in the dark (Robson et al., 2010). Therefore, further research is needed to explain how COP1 is involved in the various JA signaling pathways. A model of the regulation of JA signaling by COP1 is described in Figure 5.

## Constitutively Photomorphogenic1 Involvement in ABA Signaling

There has been little research about the link between light and ABA signals (Koniger et al., 2010; Tang et al., 2013; Fernando and Schroeder, 2015). The roles of light and ABA signals in plant physiological processes appear to be antagonistic. For example, blue light can induce stomatal opening, while ABA promotes stomatal closure (Wang et al., 2011); light can induce seed germination, while ABA inhibits it (Seo et al., 2006). Also, light promotes root growth for a short period, whereas ABA suppresses root growth (Li et al., 2017b; Liu et al., 2019). COP1 appears to be involved with these two signals (Khanna et al., 2014).

ABA-insensitive 5 (ABI5) is a bZIP transcription factor involved in ABA-mediated responses and osmotic stresses, and that plays a vital role in the ABA-induced inhibition of seed germination (Lopez-Molina and Chua, 2000; Brocard et al., 2002). The ABI5 binding protein AFP1 can promote ABI5 degradation, and their cellular levels are inversely related (Doerks et al., 2002). HY5 and BBX21, the substrates of COP1, can both regulate *ABI5* expression by binding to the *ABI5* promoter and COP1 is co-localized with ABI5 and AFP1 (Chen et al., 2008; Xu et al., 2014; Kang et al., 2018).

COP1 participates not only in ABA-regulated seed germination but also in ABA-induced stomatal closure. Decreases in COP1 reduce the ability of ABA to induce microtubule degradation, thereby making the *cop1-4* stomata insensitive to ABA in this particular response (Khanna et al., 2014). Further research is needed to explain the role of COP1 in ABA signaling and responses.

## Constitutively Photomorphogenic1 Involvement in Cytokinin Signaling

Similar to JA, cytokinins (CTKs) can promote photomorphogenesis in the dark, and thus promote hypocotyl elongation, cotyledon unfolding, and expression of light-regulated genes (Giovani et al., 2003; Vandenbussche et al., 2007b).

The effect of CTKs in photomorphogenesis may be indirect through cross-talk with other hormonal pathways (Alabadi et al., 2004). The molecular mechanisms controlling the effect of CTKs in photomorphogenesis remain unclear, but external application of CTK inhibits *COP1* transcription (Chen et al., 2014), suggesting that COP1 plays a role in the CTK-induced photomorphogenesis in the dark. JA-mediated induction of COP1 accumulation in the cytoplasm is crucial for promoting photomorphogenesis in darkness, but CTK treatment did not influence the nuclear export of GUS-COP1 in the dark, indicating that the regulatory mechanisms used by CTKs and JA are different. CTKs can also induce the accumulation of anthocyanin in darkness while COP1 function can weaken such process. Moreover, CTKs can upregulate HY5 protein levels, which are the substrates of COP1, but HY5 protein accumulation is not observed in *cop1-5* mutants (Vandenbussche et al., 2007b). Consequently, CTK may regulate some biological processes through the COP1-HY5 pathway, but the detailed mechanism remains unclear.

## Constitutively Photomorphogenic1 Involvement in Strigolactone Signaling

The strigolactone (SL) signaling pathway remains largely unknown, but it is generally accepted that there are two different pathways: a short-term reaction pathway, and a long-term reaction pathway. Typically, the short-term reaction pathway can positively regulate photomorphogenesis through the MAX2 F-box protein, and it has been suggested that MAX2 affects HY5 protein stability (Shen et al., 2012).

In the long-term pathway, SL suppresses COP1 function, resulting in HY5 accumulation and promotion of photomorphogenesis through nuclear export or degradation of COP1 (Tsuchiya et al., 2010). The SL mimic GR24 can continuously up-regulate HY5 expression both under light and in darkness, but the accumulation of HY5 protein is only dependent on the light-Phys/CRYs pathway (Jia et al., 2014). These results suggest that SL might affect the activity of COP1 to promote photomorphogenesis.

## Conclusion

The role of COP1 in light signal transduction has been firmly established over the last two decades (Osterlund et al., 1999; Li et al., 2011; Podolec and Ulm, 2018); however, recent studies have proved the additional involvement of COP1 in several plant hormonal signaling pathways (Luo et al., 2010; Liang et al., 2012; Sassi et al., 2012). As a result, COP1 has emerged as an important link in the cross-talk between light and hormonal signaling pathways. To date, there have been only few studies into the role of COP1 on the effect of light on plant growth-inducing hormones and the exact molecular mechanisms underpinning photomorphogenesis and skotomorphogenesis remain the subject of speculation. Aside from influencing hormonal signaling pathways, COP1 can also be affected by plant hormones, which can influence its nuclear accumulation, although further research is needed. Plant hormones can regulate light signal in different manners with JA, CTK, and SL promoting photomorphogenesis and ethylene, BR and GA suppressing it (Von Arnim et al., 1997; Tsuchiya et al., 2010; Zheng et al., 2017), suggesting a complex relationship with COP1.

In summary, the available data indicate that the relationship between light signals and different hormone signals is characterized by a complicated integrated signal network involving COP1. Future studies should focus on the role of COP1 in different hormone regulatory pathways within this signal network.

## Author Contributions

WW and QC wrote the manuscript paper. SG and JB revised the paper and helped in polishing the language of this article.

### Conflict of Interest Statement

The authors declare that the research was conducted in the absence of any commercial or financial relationships that could be construed as a potential conflict of interest.
